# Supplementary material for: Sleep problems were unrelated to social media use in the late COVID-19 pandemic phase: A cross-national study
Source: PLoS One. 2025 Jan 31;20(1):e0318507. doi: 10.1371/journal.pone.0318507 (PMC11785299; doi:10.1371/journal.pone.0318507)
Supplement: S1 File — (PDF) [file pone.0318507.s001.pdf]

Alle skriftlige henvendelser om saken må sendes via REK-portalen
Du finner informasjon om REK på våre hjemmesider rekportalen.no

REK
REGIONALE KOMITEER FOR MEDISINSK OG HELSEFAGLIG FORSNINGSETIKK

Region:	Saksbehandler:	Telefon:	Vår dato:	Vår referanse:
REK sør-øst B	Elin Evju Sagbakken	22845502	06.04.2020	132066
			**Deres referanse:**	

Amy K. Østertun Geirdal

132066 Opplevelse av COVID19 og koronaepidemi

Forskningsansvarlig: OsloMet - storbyuniversitetet

Søker: Amy K. Østertun Geirdal

Søkers beskrivelse av formål:

BAKGRUNNEN for studien er den pågående coronaepidemien, «nedstengingen» av Norge og pålagt sosial distansering s.
Alle over 18 år innviteres til deltagelse i prosjektet som har til hensikt å undersøke hvordan koronaepidemien med pålagt sosial distansering har sammenheng med hvordan den enkelte har det, arbeidssituasjon og bruk av sosiale medier.
Kunnskapen kan ha betydning for hvordan sosial- og helsepersonell møter personer i en slik situasjon. Forskningsprosjektet er initiert av forskere ved OsloMet.
HVA STUDIEN INNEBÆRER: Spørreskjemaet inkluderer spørsmål vedr. demografi som alder, kjønn, arbeidstilknytning, om bruk av sosiale medier og hvordan den enkelte har det, inkludert opplevelse av ensomhet, bekymring og engstelse og overordnet livskavlitet.
Det tar i underkant av 10 minutter å svare på spørsmålene.
REKRUTTERING: Prosjektet vil i sin helhet være nettbasert, med en "landingsside" på hjemmesiden til forskningsgruppen SAFE ved OsloMet, og legges ut med invitasjon og spørreskjema på ulike sosiale medier som FaceBook, LinkedIn mm. Alle over 18 år som bruker disse mediene inviteres.
Iom en ikke kjenner populasjonen er det vanskelig å si noe om representativitet
METODE: Studien er en tverrsnittstudie, og relevante statistiske analysemetoder vil bli brukt.
POTENSIELLE FORDELER OG ULEMPER:
Forskningsstudien innebærer ingen kjente potensielle ulemper utover at den enkelte

respondenten må tenke igjennom hvordan han/hun har det og hvordan hverdagen er, og bruk av sosiale medier.
HVA SOM VIL SKJE MED INFORMASJONEN:
Dataene som registreres vil bli brukt i samsvar med formålet med undersøkelsen som beskrevet. Spørreskjemaet inneholder ikke spørsmål som bidrar til at svarene kan kobles til den enkelte person, verken ved navn, fødselsnummer eller annen opplysning, som IP-adresse. Det vil ikke være mulig for forskerne å finne tilbake til den enkelte verken direkte eller indirekte eller å identifisere resultatene av studien når disse publiseres. Dette betyr at både svarene som gis og senere publisering vil være helt anonymisert.
Ved innsending av utfylt spørreskjema vil den enkelte få mulighet til å få kvittering på innsendt skjema. En må da oppgi da e-mail adresse,som kvitteringen sendes til. Din e-postadresse eller IP-adresse lagres IKKE, og kan ikke identifiseres av forskerne.
Frivillig deltagelse
FRIVILLIGHET: Å delta i undersøkelsen er frivillig. Inklusjonskriterie er at respondenten er 18 år eller eldre.
PERSONVERN OG FORSKNINGSETIKK: Den eneste informasjonen som beholdes er de anonyme svarene gitt i spørreskjemaet.
Studien skjer i henhold til anerkjente etiske forskningsetiske normer, men er, etter retningslinjer fra NSD ikke meldt NSD ettersom alle opplysninger behandles anonymt, og ikke kan spores. Studien er kvalitetssjekket og godkjent av OsloMet.
Deling av materiale og data med andre parter
Resultater kan gjøres tilgjengelig for andre forskere.
FINANSIERING OG ROLLEN TIL OSLOMET:
Studien finansieres av interne midler ved Institutt for Sosialfag og Helsefag ved OsloMet. Prosjektet ledes av professor Amy Østertun Geirdal ved institutt for Sosialfag, og er et samarbeid på tvers av fakultet ved OsloMet.
INFORMASJON OM UTFALLET AV STUDIEN
Hvis respondenten ønsker informasjon om resultatene av studien må han eller hun ta aktiv kontakt med forskerne.
Kontakt: Kontaktperson professor Amy Østertun Geirdal amyoge@oslomet.no hvis du har spørsmål og kommentarer til undersøkelsen.
INFORMERT SAMTYKKE:
Selv om studien er anonym vil den enkelte måtte svare aktivt ja på deltagelse.

REKs vurdering

Vi viser til skjema for framleggingsvurdering mottatt 03.04.2020 for prosjekt «Opplevelse av COVID19 og koronaepidemi».

Fremleggingsvurderingen er vurdert av leder i REK sør-øst B.

Slik prosjektet og dets formål vurderes, basert på de opplysninger som fremkommer av framleggingsvurderingen er hensikten å undersøke hvordan koronaepidemien med pålagt

sosial distansering har sammenheng med hvordan den enkelte har det, arbeidssituasjon og bruk av sosiale medier.

Prosjektet vil i sin helhet være nettbasert, med en "landingsside" på hjemmesiden til forskningsgruppen SAFE ved OsloMet, og legges ut med invitasjon og spørreskjema på ulike sosiale medier som FaceBook, LinkedIn mm. Alle over 18 år som bruker disse mediene inviteres. Spørreskjemaet inneholder ikke spørsmål som bidrar til at svarene kan kobles til den enkelte person, verken ved navn, fødselsnummer eller annen opplysning, som IP-adresse.

Kunnskapen kan ha betydning for hvordan sosial- og helsepersonell møter personer i en slik situasjon. Forskningsprosjektet er initiert av forskere ved OsloMet.

Etter REKs vurdering faller prosjektet, slik det er beskrevet, utenfor virkeområdet til helseforskningsloven da formålet er å kartlegge å hvordan koronaepidemien med pålagt sosial distansering har sammenheng med hvordan den enkelte har det, arbeidssituasjon og bruk av sosiale medier.

Helseforskningsloven gjelder for medisinsk og helsefaglig forskning, i loven definert som forskning på mennesker, humant biologisk materiale og helseopplysninger, som har som formål å frambringe ny kunnskap om helse og sykdom, jf. helseforskningsloven §§ 2 og 4a. Formålet er avgjørende, ikke om forskningen utføres av helsepersonell eller på pasienter/sårbare grupper eller benytter helseopplysninger.

Vedtak

Ikke fremleggspliktig

Prosjekter som faller utenfor helseforskningslovens virkeområde kan gjennomføres uten godkjenning av REK. Det er institusjonens ansvar på å sørge for at prosjektet gjennomføres på en forsvarlig måte med hensyn til for eksempel regler for taushetsplikt og personvern.

Vi gjør oppmerksom på at vurderingen og konklusjonen er å anse som veiledende jf. forvaltningsloven § 11.

Dersom dere likevel ønsker å søke REK vil søknaden bli behandlet i komitémøte, og det vil bli fattet et enkeltvedtak etter forvaltningsloven.

Vi ber om at alle henvendelser sendes inn via vår saksportal: https://rekportalen.no. Vennligst oppgi vårt referansenummer i korrespondansen.

Med vennlig hilsen

Elin Evju Sagbakken
Seniorrådgiver
REK sør-øst B
