# Supplementary material for: Sleep problems were unrelated to social media use in the late COVID-19 pandemic phase: A cross-national study
Source: PLoS One. 2025 Jan 31;20(1):e0318507. doi: 10.1371/journal.pone.0318507 (PMC11785299; doi:10.1371/journal.pone.0318507)
Supplement: S2 File — (PDF) [file pone.0318507.s002.pdf]

UNIVERSITY OF MICHIGAN

Mary Ruffolo <mruffolo@umich.edu>

A core committee has decided that no review is required for your amendment.

1 message

eresearch@umich.edu <eresearch@umich.edu> Tue, Oct 19, 2021 at 1:33 PM
Reply-To: eresearch@umich.edu
To: mruffolo@umich.edu

UNIVERSITY OF MICHIGAN
eResearch.umich.edu

Health Sciences and Behavioral Sciences Institutional Review Board (IRB-HSBS) • 2800 Plymouth Rd., Building 520, Room 1170, Ann Arbor, MI 48109-2800 • phone (734) 936-0933 • fax (734) 998-9171 • irbhsbs@umich.edu

To: Mary Ruffolo

From:

Riann Palmieri-Smith
Thad Polk

Cc:

Mary Ruffolo

Subject: No IRB Approval Required for Amendment [Ame00117522] for [HUM00180926]

SUBMISSION INFORMATION:
Study Title: The effects of social distancing on mental health, quality of life and the use of social media amid COVID-19
Full Study Title (if applicable): The effects of social distancing on mental health, quality of life and the use of social media amid the COVID-19 pandemic
Study eResearch ID: HUM00180926
Amendment eResearch ID: Ame00117522
Amendment Title: HUM00180926_Amendment - Sun Oct 17 20:18:01 EDT 2021
Date of this Notification from IRB: 10/19/2021
IRB Exemption Determination Date: 4/22/2020

UM Federalwide Assurance: FWA00004969 (For the current FWA expiration date, please visit the UM HRPP Webpage)
OHRP IRB Registration Number(s): IRB00000246

IRB EXEMPTION STATUS:
The IRB HSBS has reviewed the amendment to the study referenced above and determined that IRB approval is not required for the proposed changes.

The study, as currently described, continues to be exempt from ongoing IRB review, per the category specified on the "IRB Exemption Determination" letter on the date listed above.

Note that the study is considered exempt as long as any changes to the use of human subjects (including their data) remain within the scope of the exemption category. Any proposed changes that may exceed the scope of this category, or the approval conditions of any other non-IRB reviewing committees, must be submitted as another amendment through eResearch.

Although an exemption determination eliminates the need for ongoing IRB review and approval, you still have an obligation to understand and abide by generally accepted principles of responsible and ethical conduct of research. Examples of these principles can be found in the Belmont Report as well as in guidance from professional societies and scientific organizations.

ACCESSING EXEMPT STUDIES IN eRESEARCH:
This study must now be accessed from the "Exempt and Not Regulated" tab of the eResearch Home Workspace.

SUBMITTING AMENDMENTS VIA eRESEARCH:
You can access the online forms for amendments to this exempt study in the eResearch workspace referenced above.

TERMINATION:
You will receive an annual message reminding you of your responsibilities to manage this research application. Submit a Termination Report once you only hold or are analyzing deidentified data, or the research has ended.

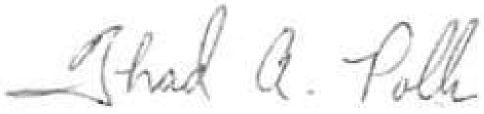

Riann Palmieri-Smith **Thad Polk**
Co-chair, IRB HSBS **Co-chair, IRB HSBS**
