# Supplementary material for: Sleep problems were unrelated to social media use in the late COVID-19 pandemic phase: A cross-national study
Source: PLoS One. 2025 Jan 31;20(1):e0318507. doi: 10.1371/journal.pone.0318507 (PMC11785299; doi:10.1371/journal.pone.0318507)
Supplement: S3 File — (PDF) [file pone.0318507.s003.pdf]

Notification of Exemption from Human Research Ethics Review

**Project Number:**	2021/HE002544
**Project Title:**	The Impact of Covid-19 social distancing on mental Health and Quality of Life. A cross-national comparative study
**Version:**	0.01
**Chief Investigator:**	Dr Janni King Yi Leung<br>National Centre for Youth Substance Use Research
**Co-Investigator(s)**	Dr Amy Ostertun Geirdal<br>Ms Daica Price<br>Dr Gary Lamph<br>Dr Hilde Thygesen<br>Dr Mariyana Schloutz<br>Ms Caitlin MCCLURE-THOMAS
**Exemption End Date:**	31 Dec 2022
**Date of Exemption:**	Tuesday, 16 November 2021

Data Source:

n/a

This project has been reviewed by the Research Ethics and Integrity and is deemed to be exempt from ethics review under the National Statement on Ethical Conduct in Human Research and relevant University of Queensland policy (PPL 4.20.07).

Delegated Officer
Research Ethics and Integrity
The University of Queensland
