# Supplementary material for: Sleep problems were unrelated to social media use in the late COVID-19 pandemic phase: A cross-national study
Source: PLoS One. 2025 Jan 31;20(1):e0318507. doi: 10.1371/journal.pone.0318507 (PMC11785299; doi:10.1371/journal.pone.0318507)
Supplement: S4 File — (PDF) [file pone.0318507.s004.pdf]

University of Central Lancashire
UCLan

University of Central Lancashire
Preston PR1 2HE
01772 201201
uclan.ac.uk

12 November 2021

Gary Lamph
School of Nursing
University of Central Lancashire

Dear Gary

Re: Health Ethics Review Panel Application
Unique reference Number: HEALTH 0246

The Health Ethics Review Panel has granted approval of your proposal application 'The effects of social distancing on mental health, quality of life and the use of social media amid the COVID-19 pandemic'. Approval is granted up to the end of project date*. It is your responsibility to ensure that

- the project is carried out in line with the information provided in the forms you have submitted
- you regularly re-consider the ethical issues that may be raised in generating and analysing your data
- any proposed amendments/changes to the project are raised with, and approved by, the Ethics Review Panel
- you notify EthicsInfo@uclan.ac.uk if the end date changes or the project does not start
- serious adverse events that occur from the project are reported to the Ethics Review Panel
- a closure report is submitted to complete the ethics governance procedures (existing paperwork can be used for this purpose e.g. funder's end of grant report; abstract for student award or NRES final report. If none of these are available use e-Ethics Closure Report pro forma).

Please also note that it is the responsibility of the applicant to ensure that the ethics committee that has already approved this application is either run under the auspices of the National Research Ethics Service or is a fully constituted ethics committee, including at least one member independent of the organisation or professional group.

Yours sincerely

Jane Fitzgerald
Deputy Vice Chair
Health Ethics Review Panel
