# Supplementary material for: Sleep problems were unrelated to social media use in the late COVID-19 pandemic phase: A cross-national study
Source: PLoS One. 2025 Jan 31;20(1):e0318507. doi: 10.1371/journal.pone.0318507 (PMC11785299; doi:10.1371/journal.pone.0318507)
Supplement: S5 File — (PDF) [file pone.0318507.s005.pdf]

Amy K. Østertun Geirdal
132066 Experience of COVID-19 and the Corona Epidemic
Responsible for Research: OsloMet - Oslo Metropolitan University
Applicant: Amy K. Østertun Geirdal

Applicant's description of the purpose:

The **BACKGROUND** of the study is the ongoing coronavirus epidemic, the "lockdown" of Norway, and the enforced social distancing.
Everyone over 18 years old is invited to participate in the project, which aims to examine how the coronavirus epidemic and the enforced social distancing relate to individuals' well-being, work situation, and use of social media. The knowledge gained could be important for how social and healthcare professionals address people in such situations. The research project is initiated by researchers at OsloMet.

WHAT THE STUDY INVOLVES: The questionnaire includes questions related to demographics such as age, gender, employment status, social media usage, and individual well-being, including experiences of loneliness, worry, anxiety, and overall life quality. It takes less than 10 minutes to answer the questions.

RECRUITMENT: The project will be entirely online, with a "landing page" on the homepage of the SAFE research group at OsloMet. The invitation and questionnaire will be distributed via various social media platforms such as Facebook, LinkedIn, etc. Everyone over 18 years old who uses these media is invited. Since the population is unknown, it is difficult to comment on representativeness.

METHOD: The study is a cross-sectional study, and relevant statistical analysis methods will be used.

POTENTIAL BENEFITS AND DRAWBACKS: The research study involves no known potential drawbacks other than that respondents must reflect on how they are doing, their daily lives, and their use of social media.

WHAT WILL HAPPEN TO THE INFORMATION: The data collected will be used according to the purpose of the study as described. The questionnaire does not contain questions that can link responses to individuals, either by name, personal identification number, or other information such as IP addresses. It will not be possible for the researchers to trace back to any individual directly or indirectly or to identify the study results when published. This means that both the responses provided and subsequent publications will be completely anonymized.

Upon submitting the completed questionnaire, participants will have the opportunity to receive a receipt for submission. To receive the receipt, they must provide their email address, to which the receipt will be sent. Neither the email address nor the IP address will be stored and cannot be identified by the researchers.

VOLUNTARY PARTICIPATION
Participation in the survey is voluntary. The inclusion criterion is that the respondent is 18 years or older.

PRIVACY AND RESEARCH ETHICS: The only information retained will be the anonymous responses given in the questionnaire. The study adheres to recognized ethical research norms, but it is not reported to the NSD (Norwegian Centre for Research Data), as all information is

processed anonymously and cannot be traced. The study has been quality-checked and approved by OsloMet.

Sharing of material and data with other parties:
Results may be made available to other researchers.

FUNDING AND THE ROLE OF OSMOLET:
The study is funded by internal funds at the Department of Social and Health Sciences at OsloMet. The project is led by Professor Amy Østertun Geirdal at the Department of Social Sciences and is a cross-faculty collaboration at OsloMet.

INFORMATION ON THE OUTCOME OF THE STUDY:
If respondents want information on the study's results, they must actively contact the researchers.

Contact: Contact person: Professor Amy Østertun Geirdal at amyoge@oslomet.no if you have any questions or comments regarding the survey.

INFORMED CONSENT:
Although the study is anonymous, participants must actively agree to participate.

REK's Evaluation:
We refer to the submission evaluation form received on 03.04.2020 for the project "Experience of COVID-19 and the Corona Epidemic." The evaluation has been reviewed by the leader of REK South-East B. Based on the description provided, the project aims to investigate how the corona epidemic and enforced social distancing are related to individual well-being, work situation, and social media usage. The project will be entirely online, with a landing page on the research group SAFE's homepage at OsloMet, and will be shared on various social media platforms such as Facebook, LinkedIn, etc. Everyone over 18 years old who uses these platforms is invited. The questionnaire does not contain questions that can link responses to individuals, either by name, personal identification number, or other information such as IP addresses. The knowledge gained could be important for how social and healthcare professionals address people in such situations. The research project is initiated by researchers at OsloMet.

Decision:
Not subject to presentation requirement.
Projects that fall outside the scope of the Health Research Act may be conducted without REK approval. It is the institution's responsibility to ensure that the project is carried out in a responsible manner, for example, in accordance with confidentiality and privacy regulations.

We note that the assessment and conclusion should be considered as guidance according to Section 11 of the Public Administration Act. Should you wish to apply to REK, the application will be considered in a committee meeting, and an individual decision will be made in accordance with the Public Administration Act.

We ask that all inquiries be submitted via our case portal: https://rekportalen.no.
Please include our reference number in the correspondence.

Kind regards,
Elin Evju Sagbakken
Senior Advisor, REK South-East
