# Supplementary material for: Sleep problems were unrelated to social media use in the late COVID-19 pandemic phase: A cross-national study
Source: PLoS One. 2025 Jan 31;20(1):e0318507. doi: 10.1371/journal.pone.0318507 (PMC11785299; doi:10.1371/journal.pone.0318507)
Supplement: S6 File — (PDF) [file pone.0318507.s006.pdf]

To whom it may concern

**Date:** 26. October 2021 **Our ref.:** 20/03676

**Executive officer:** name
**Exempt from public:**

# Approval of project «The impact of COVID-19 social distancing on mental Health and Quality of life. A cross-national comparative study. *Survey 3;* COVID-19 Wellness Survey", project leader professor Amy Østertun Geirdal.

The Survey will have a landing site in each participating country; USA (University of Michigan, Ann Arbor (prof. Mary Ruffolo)), UK (University of Central Lancashire (dr. Gary Lamph)), Australia (The University of Queensland (dr. Janni Liung) and Norway (OsloMet).

All data will be stored at "nettskjema" (a web form) directed by OsloMet. All approvals for collecting and storing data are met according to Norwegian rules and laws.

The project has been approved by Oslo Metropolitan University (approval number 20/03676) and will not process any personal data.

We also refer to the assessment by the regional Committees for medical and health research ethics, number 132066

Inger Flatland

Inger Johanne Flatland
GDPR contact person at
the Faculty of Social Sciences


**OSLO METROPOLITAN UNIVERSITY**
STORBYUNIVERSITETET

**P.O. Box**
Pb. 4 St. Olavs plass, NO-0130 OSLO

**Insert organisational unit**

**Tel.**
+ 47 67 23 50 00

**Visiting address**
Pilestredet 46
