# Supplementary material for: Sleep problems were unrelated to social media use in the late COVID-19 pandemic phase: A cross-national study
Source: PLoS One. 2025 Jan 31;20(1):e0318507. doi: 10.1371/journal.pone.0318507 (PMC11785299; doi:10.1371/journal.pone.0318507)
Supplement: Attachment — Submitted filename: Response to reviewers.docx [file pone.0318507.s007.docx]

Authors: Thank you for your comments and suggestions, which we believe have contributed to strengthening the article. The revised manuscript shows all changes tracked. Each comment has been responded to point by point in this letter. We look forward to hearing from you again.

Editor: Dear Dr. Bonsaksen, thank you for submitting your manuscript to PLOS ONE. After careful consideration, we feel that it has merit but does not fully meet PLOS ONE’s publication criteria as it currently stands. Therefore, we invite you to submit a revised version of the manuscript that addresses the points raised during the review process.

Authors: Thank you.

Editor: Please submit your revised manuscript by Feb 09 2025 11:59PM. If you will need more time than this to complete your revisions, please reply to this message or contact the journal office at [plosone@plos.org](mailto:plosone@plos.org). When you're ready to submit your revision, log on to <https://www.editorialmanager.com/pone/> and select the 'Submissions Needing Revision' folder to locate your manuscript file.

Please include the following items when submitting your revised manuscript:

- A rebuttal letter that responds to each point raised by the academic editor and reviewer(s). You should upload this letter as a separate file labeled 'Response to Reviewers'.

Authors: This letter is the rebuttal letter.

Editor:

- A marked-up copy of your manuscript that highlights changes made to the original version. You should upload this as a separate file labeled 'Revised Manuscript with Track Changes'.
- An unmarked version of your revised paper without tracked changes. You should upload this as a separate file labeled 'Manuscript'.

Authors: Both files have been labeled according to this requirement.

Editor: If you would like to make changes to your financial disclosure, please include your updated statement in your cover letter. Guidelines for resubmitting your figure files are available below the reviewer comments at the end of this letter.

Authors: We have included our financial disclosure in the cover letter. No figure files have been used.

Editor: If applicable, we recommend that you deposit your laboratory protocols in protocols.io to enhance the reproducibility of your results. Protocols.io assigns your protocol its own identifier (DOI) so that it can be cited independently in the future. For instructions see: [https://journals.plos.org/plosone/s/submission-guidelines#loc-laboratory-protocols](https://eur04.safelinks.protection.outlook.com/?url=https%3A%2F%2Ftrack.editorialmanager.com%2FCL0%2Fhttps%3A%252F%252Fjournals.plos.org%252Fplosone%252Fs%252Fsubmission-guidelines%2523loc-laboratory-protocols%2F1%2F010f0194033385d9-bc675491-563b-49ad-aeee-f63e896e83c7-000000%2FsLXanRwvaHgCHntOUQc7OaEjfclanD5h84omx_nWPLo%3D191&data=05%7C02%7Ctore.bonsaksen%40inn.no%7C8a2b6772a9664044034f08dd25b30598%7Cd45cfa45904a497297413335dc0372b4%7C0%7C0%7C638708173322389543%7CUnknown%7CTWFpbGZsb3d8eyJFbXB0eU1hcGkiOnRydWUsIlYiOiIwLjAuMDAwMCIsIlAiOiJXaW4zMiIsIkFOIjoiTWFpbCIsIldUIjoyfQ%3D%3D%7C0%7C%7C%7C&sdata=LbXooo8HfM22WTGD5PxkELAgkeCf4JrQP8mnvLuktRk%3D&reserved=0). Additionally, PLOS ONE offers an option for publishing peer-reviewed Lab Protocol articles, which describe protocols hosted on protocols.io. Read more information on sharing protocols at [https://plos.org/protocols?utm_medium=editorial-email&utm_source=authorletters&utm_campaign=protocols](https://eur04.safelinks.protection.outlook.com/?url=https%3A%2F%2Ftrack.editorialmanager.com%2FCL0%2Fhttps%3A%252F%252Fplos.org%252Fprotocols%253Futm_medium%3Deditorial-email%2526utm_source%3Dauthorletters%2526utm_campaign%3Dprotocols%2F1%2F010f0194033385d9-bc675491-563b-49ad-aeee-f63e896e83c7-000000%2FjzMVGI6dx7mLAQh-cq0N3qKiG7lw0vkOk4BuCwP4Tow%3D191&data=05%7C02%7Ctore.bonsaksen%40inn.no%7C8a2b6772a9664044034f08dd25b30598%7Cd45cfa45904a497297413335dc0372b4%7C0%7C0%7C638708173322406479%7CUnknown%7CTWFpbGZsb3d8eyJFbXB0eU1hcGkiOnRydWUsIlYiOiIwLjAuMDAwMCIsIlAiOiJXaW4zMiIsIkFOIjoiTWFpbCIsIldUIjoyfQ%3D%3D%7C0%7C%7C%7C&sdata=kUPmHg8S%2B1TXDwlZm3IMRS99CUQP%2FhLMahIXcDjVxSY%3D&reserved=0).

Authors: No laboratory protocols have been used.

Editor: Journal Requirements: When submitting your revision, we need you to address these additional requirements.

1. Please ensure that your manuscript meets PLOS ONE's style requirements, including those for file naming. The PLOS ONE style templates can be found at

<https://journals.plos.org/plosone/s/file?id=wjVg/PLOSOne_formatting_sample_main_body.pdf> and 
<https://journals.plos.org/plosone/s/file?id=ba62/PLOSOne_formatting_sample_title_authors_affiliations.pdf>

Authors: We have consulted the relevant documents and have modified the files accordingly.

Editor: 2. Please include a complete copy of PLOS’ questionnaire on inclusivity in global research in your revised manuscript. Our policy for research in this area aims to improve transparency in the reporting of research performed outside of researchers’ own country or community. The policy applies to researchers who have travelled to a different country to conduct research, research with Indigenous populations or their lands, and research on cultural artefacts. The questionnaire can also be requested at the journal’s discretion for any other submissions, even if these conditions are not met.

Please find more information on the policy and a link to download a blank copy of the questionnaire here: <https://journals.plos.org/plosone/s/best-practices-in-research-reporting>.

Please upload a completed version of your questionnaire as Supporting Information when you resubmit your manuscript.

Authors: As the policy applies to researchers who have travelled to a different country to conduct their research, it does not apply to this manuscript. The research group (including those acknowledged) represent all of the countries where data collection took place in this study.

Editor: 3. We note that the grant information you provided in the ‘Funding Information’ and ‘Financial Disclosure’ sections do not match.

When you resubmit, please ensure that you provide the correct grant numbers for the awards you received for your study in the ‘Funding Information’ section.

Authors: We would like the statement to read as follows: “Funding to cover the APC for the article was granted by University of Inland Norway. Otherwise, no funding was obtained for this study.” However, note two issues: There is no grant number for the financial support we received to cover the APC for this study. In addition, my university recently changed its name from Inland Norway University of Applied Sciences to University of Inland Norway. The latter is not yet possible to select from the list of funders, thus, discrepancies may still be found related to statements of funding.

Editor: 4. Please note that funding information should not appear in the Acknowledgments section or other areas of your manuscript. We will only publish funding information present in the Funding Statement section of the online submission form. Please remove any funding-related text from the manuscript.

Authors: Removed as required.

Editor: 5. We note that you have indicated that there are restrictions to data sharing for this study. For studies involving human research participant data or other sensitive data, we encourage authors to share de-identified or anonymized data. However, when data cannot be publicly shared for ethical reasons, we allow authors to make their data sets available upon request. For information on unacceptable data access restrictions, please see <http://journals.plos.org/plosone/s/data-availability#loc-unacceptable-data-access-restrictions>.

Authors: Please refer to our response to Editor comment no. 6.

Editor: Before we proceed with your manuscript, please address the following prompts:

a) If there are ethical or legal restrictions on sharing a de-identified data set, please explain them in detail (e.g., data contain potentially identifying or sensitive patient information, data are owned by a third-party organization, etc.) and who has imposed them (e.g., a Research Ethics Committee or Institutional Review Board, etc.). Please also provide contact information for a data access committee, ethics committee, or other institutional body to which data requests may be sent.

b) If there are no restrictions, please upload the minimal anonymized data set necessary to replicate your study findings to a stable, public repository and provide us with the relevant URLs, DOIs, or accession numbers. Please see <http://www.bmj.com/content/340/bmj.c181.long> for guidelines on how to de-identify and prepare clinical data for publication. For a list of recommended repositories, please see <https://journals.plos.org/plosone/s/recommended-repositories>. You also have the option of uploading the data as Supporting Information files, but we would recommend depositing data directly to a data repository if possible.

Please update your Data Availability statement in the submission form accordingly.

6. In this instance it seems there may be acceptable restrictions in place that prevent the public sharing of your minimal data. However, in line with our goal of ensuring long-term data availability to all interested researchers, PLOS’ Data Policy states that authors cannot be the sole named individuals responsible for ensuring data access (<http://journals.plos.org/plosone/s/data-availability#loc-acceptable-data-sharing-methods>).

Data requests to a non-author institutional point of contact, such as a data access or ethics committee, helps guarantee long term stability and availability of data. Providing interested researchers with a durable point of contact ensures data will be accessible even if an author changes email addresses, institutions, or becomes unavailable to answer requests.

Before we proceed with your manuscript, please also provide non-author contact information (phone/email/hyperlink) for a data access committee, ethics committee, or other institutional body to which data requests may be sent. If no institutional body is available to respond to requests for your minimal data, please consider if there any institutional representatives who did not collaborate in the study, and are not listed as authors on the manuscript, who would be able to hold the data and respond to external requests for data access? If so, please provide their contact information (i.e., email address). Please also provide details on how you will ensure persistent or long-term data storage and availability.

Authors: We have modified our position on this matter, and we have provided access to the minimal dataset allowing for the replication of the analyses performed in the study. The data can be accessed from here: https://doi.org/10.18710/B69ATB. See also our modified data availability statement in the manuscript.

Editor: 7.  Your ethics statement should only appear in the Methods section of your manuscript. If your ethics statement is written in any section besides the Methods, please delete it from any other section.

Authors: Removed from all other sections.

Editor: 8. Please include captions for your Supporting Information files at the end of your manuscript, and update any in-text citations to match accordingly. Please see our Supporting Information guidelines for more information: <http://journals.plos.org/plosone/s/supporting-information>.

Authors: Captions for supplementary files have been included at the end of the manuscript, and the uploaded supplementary files have been labeled according to the guidelines.

Editor: Dear authors, Three reviewers have evaluated your investigation. Please, respond to all their comments so we can reach a final decision after carefully considering your responses.

Authors: All comments have been responded to in this letter.

Reviewers:
1. Is the manuscript technically sound, and do the data support the conclusions?

The manuscript must describe a technically sound piece of scientific research with data that supports the conclusions. Experiments must have been conducted rigorously, with appropriate controls, replication, and sample sizes. The conclusions must be drawn appropriately based on the data presented.

Reviewer #1: Yes

Reviewer #2: Partly

Reviewer #3: Yes

Authors: Please refer to our responses to the specific reviewer comments.

2. Has the statistical analysis been performed appropriately and rigorously?

Reviewer #1: Yes

Reviewer #2: No

Reviewer #3: Yes

Authors: Please refer to our responses to the specific reviewer comments.

3. Have the authors made all data underlying the findings in their manuscript fully available?

The [PLOS Data policy](https://eur04.safelinks.protection.outlook.com/?url=http%3A%2F%2Ftrack.editorialmanager.com%2FCL0%2Fhttp%3A%252F%252Fwww.plosone.org%252Fstatic%252Fpolicies.action%2523sharing%2F1%2F010f0194033385d9-bc675491-563b-49ad-aeee-f63e896e83c7-000000%2F5wTSG1NCzdXsi5XDkyuJ0BGZDxkmGCwB5oq6IBR5Zb8%3D191&data=05%7C02%7Ctore.bonsaksen%40inn.no%7C8a2b6772a9664044034f08dd25b30598%7Cd45cfa45904a497297413335dc0372b4%7C0%7C0%7C638708173322416499%7CUnknown%7CTWFpbGZsb3d8eyJFbXB0eU1hcGkiOnRydWUsIlYiOiIwLjAuMDAwMCIsIlAiOiJXaW4zMiIsIkFOIjoiTWFpbCIsIldUIjoyfQ%3D%3D%7C0%7C%7C%7C&sdata=Z4v59tb4rUAnJPnoQvpXxEkrBu7oRkC5a02sbe3G6U8%3D&reserved=0) requires authors to make all data underlying the findings described in their manuscript fully available without restriction, with rare exception (please refer to the Data Availability Statement in the manuscript PDF file). The data should be provided as part of the manuscript or its supporting information, or deposited to a public repository. For example, in addition to summary statistics, the data points behind means, medians and variance measures should be available. If there are restrictions on publicly sharing data—e.g. participant privacy or use of data from a third party—those must be specified.

Reviewer #1: Yes

Reviewer #2: No

Reviewer #3: No

Authors: Please refer to our responses to the specific reviewer comments. Also, note that we have deposited the minimal dataset to a public repository.

4. Is the manuscript presented in an intelligible fashion and written in standard English?

PLOS ONE does not copyedit accepted manuscripts, so the language in submitted articles must be clear, correct, and unambiguous. Any typographical or grammatical errors should be corrected at revision, so please note any specific errors here.

Reviewer #1: Yes

Reviewer #2: Yes

Reviewer #3: Yes

5. Review Comments to the Author

Reviewer #1: The article stands out for its quality of exposition. The analysis of the questionnaire is conducted rigorously and contributes to developing a homogeneous and coherent argument in line with the research question formulated in the paper. However, I would suggest emphasizing the gender differences in the text, as the sample shows a significant imbalance in this area.

Authors: We agree that the gender differences are important, and we would like to refer to the Discussion section where two full paragraphs are devoted to this issue (note that the numbered references below have changed when copying the text into the new document):

“Noteworthy associations with sleep problems were shown for female gender and psychological distress, and there is reason to suggest that these factors might also explain the results for social media use. The higher levels of psychological distress and sleep problems among women has been well documented, both prior to and during the COVID-19 pandemic [1-3]. Studies have also pointed to girls and women as more frequent users of social media, compared to boys and men [4-6], and also that the association between higher social media use and lower wellbeing is stronger for girls [5]. Thus, if adult women use social media more than men and also respond to their social media experiences with more distress, this may explain why the association between social media use and sleep problems was weakened and no longer statistically significant after controlling for gender and psychological distress.

While female gender and higher psychological distress are linked, the multivariate model showed that both of these variables had a unique relationship with sleep problems. Thus, more sleep problems among women than men may partially, but not fully, be explained with reference to higher psychological distress levels among women. The processes driving sleep problems may therefore concern a range of other factors than psychological distress. Previous research has suggested biological factors such as differences in sex steroids [7] and women’s menstruational cycle [8], but women may also be psychologically more prone to lie awake pondering about things, without necessarily feeling distressed. As we do not have data to explore these possibilities further, we suggest that future research may address the processes driving sleep problems in a gender perspective.”

R1: Additionally, I would recommend updating the literature section with more recent references and including a brief paragraph that informs the reader about the current line of research exploring the link between social media addictions (such as Instagram, TikTok, and WhatsApp) and difficulties in falling asleep.

Authors: The introduction section has been updated with more materials and references concerning social media addiction and sleep problems.

Reviewer #2: This research explored the association between social media use and sleep problems approximately two years after the outbreak of the COVID-19 pandemic through a web-based survey. However, the significance of this research is unclear due to the limited information provided about the main variables, namely sleep problems and social media use. Below, I have outlined specific concerns for each section.

Authors: Please see our responses to the specific concerns listed below.

R2: METHODS
λ The authors should explain the social circumstances (e.g., restrictions) in each country where the survey was conducted during the research period. Additionally, the appropriateness of the survey period (November–January) should be considered, as social media use might increase, and physical activity might decrease during the winter season (except in Australia).

Authors: More general information about the pandemic restrictions at the time is presented (see Methods/Design section).

R2: λ Sampling bias might be present, as the participants were recruited through social media. This could mean the study sample was limited to active social media users.

Authors: We agree, and the issue has been added to the limitations section.

R2: λ The validity of the sleep problem question is questionable. The question provides only limited information about sleep issues, despite there being many types of sleep problems (e.g., insomnia, delayed sleep phase, sleep deficit).

Authors: Again, we agree, and we have specified that the sleep problems measure is not optimal and has unknown psychometric properties (see limitations section). However, we still believe there is value in asking about ‘sleep problems’ in general, without always needing to specify the types of problems.

R2: Similarly, the question on social media use also appears limited in scope. The authors mentioned in the introduction that motives for social media use may be more relevant to health outcomes than the actual time spent using them, but no data on these motives were collected.

Authors: We agree that the social media measure solely reflects time use, and that other aspects of social media use have not been included. This has been noted as part of the study limitations.

R2: RESULTS
λ The means of time on social media (4.4 and 4.1) is unclear. Does it mean 4.4 hours? Given the response option, it is not thought to be continuous value. If so, calculating average is not appropriate.

Authors: 4.4 is the calculated average on the given response scale (1-6). While we agree that 4.4 cannot be translated into a specific amount of time, it serves the purpose of providing an indication about the level of social media use in the sample (i.e., on average, the participants used social media between 1 and 3 hours on a typical day).

R2: λ The data were collected from four countries, where the seasons and pandemic circumstances are different between the countries. This factor should be considered for analysis.

Authors: In retrospect we have analyzed between-country differences in proportions of participants with reported sleep problems. Proportions were between 58.5% (USA) and 67.7% (UK), and the differences were not statistically significant. These results are reported in Table 1.

Reviewer #3: * General Aspects
We appreciate the opportunity to review this article because it is, in fact, a very relevant topic. Regarding originality, it can be considered that the article does not make a particularly relevant contribution in this field of study, although it proposes to analyze the relationship between the use of social networks, sleep problems and mental health in the adult population in general, in the context of the covid-19 pandemic. The manuscript is written in a clear and coherent way, and the theme is very important, especially if we consider that technology and social networks are part of our days and can greatly affect our well-being and quality of life. Therefore, here are some aspects that may strengthen the arguments presented and improve the article.

Authors: Thank you.

R3: *Title:
It is consistent with the content presented, clear and objective. It arouses the reader's interest, because it is a cross-national study.

Authors: Thank you.

R3: *Summary:
The abstract is clear and well organized, following the structure of the article. The introduction can, however, be improved by deepening the relationship between the use of social networks, sleep problems and mental health in the adult population in a pandemic context. It may also be useful to include more clearly the contribution/implications of the study for research in this area.

Authors: The introductory and concluding sections of the abstract have incorporated these elements as suggested.

R3: *Introduction:
The introduction adequately presents the context and importance of the study. It is clearly worded, but perhaps it is a little shorter than desirable in terms of existing research on (1) people's motivations for using social media and (2) personal experience of using it. As the authors point out, these can be very relevant aspects for mental health outcomes (even more than the time spent using social networks).

Authors: These aspects of social media use are briefly mentioned, but given the accessible data material (which does not include these aspects) we believe the narrative is more coherent as it currently stands. Including more introductory materials on aspects that we will not pursue further in formal analysis will, in our opinion, rather serve to disrupt the internal logic of the study.

R3: It is important that the introduction situates the existing knowledge on the topic under study, helping to formulate the hypotheses/arguments that will be developed in the article. There is also a need to make a link between the previous work and this article, so it would be interesting to explore further why the approach adopted is innovative. In other words, it could be useful to clarify the originality and relevance of the study, since the innovative nature of the study is not entirely clear.

Authors: While we wholeheartedly agree that the introduction should describe ‘existing knowledge on the topic under study’ and ‘make a link between the previous work and this article’, it is more difficult for us to understand what the reviewer feels is missing. We have clarified the originality and relevance of the study towards the end of the introduction section:

“Considering the evidence, the increased use of social media may give rise to more sleep problems among people. However, while the literature on associations between social media use, sleep problems, and poorer mental health is substantial, many of these studies have focused on children and adolescents [e.g., 9, 10-12]. Moreover, these associations may be different in a pandemic context where a range of other factors and events can give cause for sleep problems. The gap in the literature concerning these associations among the general adult population, and during the extraordinary context of the COVID-19 pandemic, constitutes the rationale for this study.”

R3: It would also be appropriate for the study to have a theoretical perspective that would allow a reading and interpretation of the results obtained. The study seems atheoretical.

Authors: We have included a brief description of the uses and gratifications theory that can serve as a theoretical vantage point for understanding social media use; see first page of the introduction. However, note that it will be difficult to interpret the findings (i.e., the lack of association between social media use and sleep problems) in view of any theory.

R3: The introduction ends with the presentation of the purpose of the study – which is very appropriate.

Authors: Thank you.

R3: *Methodology:
The authors adequately describe how they obtained the data, and the methodology used to analyze them. There are some reservations about the measures used to assess the daily use of social networks (using only one question to assess the amount of time people spent on social networks during a typical day during the last month can be reductive), not least because it would have been very interesting to also evaluate people's motivations for using social networks and personal experience of using it.

Authors: There are many aspects of social media use, and we fully agree with the reviewer that also motivations and experiences are important. Therefore, our study focusing on time spent on social media assesses only one aspect of the phenomenon. We have emphasized this issue in the limitations section.

R3: In addition, I did not understand exactly the purpose of questioning whether the person was infected with covid-19.

Authors: We asked whether the person had been infected with COVID-19. Previous studies have shown that COVID-19 infection, and also long-term consequences of having been infected, have included mental health problems in general, and also sleep disturbances more specifically [13]. Thus, infection status was relevant to include in our study as a covariate.

R3: Finally, from a methodological point of view, the fact that data collection took place approximately two years after the pandemic outbreak may have greatly affected the results obtained.

Authors: We have specified that the study is situated in the late pandemic phase (see e.g. title and Methods section) and make no claim that the results are valid across time.

R3: From the point of view of the characteristics of the participants, and in the case of a cross-national study, it may make sense to present a brief characterization of the participants by country as this can be useful to understand the results obtained. As regards the sample size, it is considered an acceptable size.

Authors: A new paragraph has been inserted first in the Results section, in which between-country differences on sociodemographic variables are presented in a narrative.

R3: *Results
The results section is interesting and focuses on the essentials. However, the results obtained do not seem to add anything very relevant to research in this field. In addition, the pandemic context seems not to have been considered or had no effect on the results obtained.

Authors: What is considered relevant to the research field is perhaps a matter of opinion. For our part, we believe the results showing no association between magnitude of social media use and self-reported sleep problems is relevant to the field where many studies focus on the problematic outcomes of social media use. Thus, our study offers nuance to the assessment of what possible outcomes are related to social media use. We disagree that the pandemic context is not considered, as it is considered and stated explicitly already in the title of the manuscript. However, it is considered as context for the study, and not as a comparison. Given the cross-sectional study design with only one data collection, we cannot speak of the pandemic as having an effect on the results. Rather, the results were produced in this given context.

R3: The results of the logistic regression show that time spent on social networks is not statistically associated with sleep problems, nor are there differences between men and women. That said, the question that arises is whether the results obtained are relevant to the advancement of knowledge in the field?

Authors: The results showed no association between time on social media and sleep problems, but also that women were 69% more likely than men to report sleep problems (see results section, Table 2, and Discussion). Concerning relevance to the field, we refer to our response to the preceding comment.

R3: *Discussion
The discussion begins with a synthesis of the main results, which is appropriate. There is room, however, for a deeper discussion about the results obtained because some arguments would make more sense in the introduction of the article and not in the discussion. At this point, one would expect a more in-depth critical analysis of the main results of the study. It is suggested, therefore, a greater robustness of the theoretical and empirical framework in the introduction so that in the discussion it is possible to adopt a more analytical and critical lens on the results obtained.

Authors: In principle, there is always room for more discussion, and we agree with the reviewer that the Discussion section is the appropriate place for an in-depth critical discussion of the results. However, given that we have indeed discussed all significant findings (i.e., the relations between sleep problems and age, gender, employment, and psychological distress) in addition to the main focus of the study (i.e., the relation between sleep problems and social media use), it is difficult to understand what the reviewer feels is missing. More specific guidance would be needed to enable us to address any outstanding issues with the Discussion section.

R3: Regarding the main limitations of the study, the composition of the sample may be a weakness (the fact that it includes mainly people of young age and more educated) and this has, as mentioned by the authors, implications for the generalization of the results. Likewise, it would have been useful to use more robust and complete data collection procedures/tools to explore other possibilities in data analysis.

Authors: We believe all of the mentioned issues have been appropriately discussed in the limitations section of the manuscript.

R3: It could also be useful to analyze cultural influence as this is a cross-national study.
It is also essential to make a clear statement about the contribution and/or implications of the study in relation to existing knowledge. What are, in fact, the main conclusions of the study and what implications do they have for the advancement of knowledge in this field?

Authors: In the revised manuscript, we have reported no significant between-country differences on sleep problems (Table 1). The main conclusions from the study and our assessment of what is needed to advance research in the field can be found in the Conclusion section. The contributions of the study in relation to existing knowledge can be found towards the end of the Introduction section. Reiterated here: “Considering the evidence, the increased use of social media may give rise to more sleep problems among people. However, while the literature on associations between social media use, sleep problems, and poorer mental health is substantial, many of these studies have focused on children and adolescents [e.g., 9, 10-12]. Moreover, these associations may be different in a pandemic context where a range of other factors and events can give cause for sleep problems. The gap in the literature concerning these associations among the general adult population, and during the extraordinary context of the COVID-19 pandemic, constitutes the rationale for this study”.

R3:*Conclusion
Based on the results of the study, it is concluded that the time spent on social media was not related to people's sleep problems during the final phase of the COVID-19 pandemic. Considering that this result does not meet expectations (as mentioned by the authors), it is important to deepen the study and analyze the complexity associated with the use of social networks (e.g., time of day, content of interactions, associated stress experience) regarding the sleep patterns and problems of adults.

Authors: We agree that these suggestions are important to advance knowledge in this field. However, we are unable to do so in the current study as we do not have the required data (e.g., time of day, content of interactions, associated stress experience) available.

References used in the response to reviewers:

1.	Chow CM, Homa J, Amersdorfer A. Gender differences in sleep problems: The mediating role of co-rumination and depressive symptoms. Personality and Individual Differences. 2017;108:10-3. doi: <https://doi.org/10.1016/j.paid.2016.11.058>.

2.	Ding X, Brazel DM, Mills MC. Gender differences in sleep disruption during COVID-19: cross-sectional analyses from two UK nationally representative surveys. BMJ open. 2022;12(4):e055792. doi: 10.1136/bmjopen-2021-055792.

3.	Viertiö S, Kiviruusu O, Piirtola M, Kaprio J, Korhonen T, Marttunen M, et al. Factors contributing to psychological distress in the working population, with a special reference to gender difference. BMC Public Health. 2021;21(1):611. doi: 10.1186/s12889-021-10560-y.

4.	Kimbrough AM, Guadagno RE, Muscanell NL, Dill J. Gender differences in mediated communication: Women connect more than do men. Computers in Human Behavior. 2013;29(3):896-900. doi: <https://doi.org/10.1016/j.chb.2012.12.005>.

5.	Twenge JM, Martin GN. Gender differences in associations between digital media use and psychological well-being: Evidence from three large datasets. Journal of Adolescence. 2020;79:91-102. doi: <https://doi.org/10.1016/j.adolescence.2019.12.018>.

6.	Scott CF, Bay-Cheng LY, Prince MA, Nochajski TH, Collins RL. Time spent online: Latent profile analyses of emerging adults' social media use. Computers in Human Behavior. 2017;75:311-9. doi: <https://doi.org/10.1016/j.chb.2017.05.026>.

7.	Mong JA, Cusmano DM. Sex differences in sleep: impact of biological sex and sex steroids. Philosophical Transactions of the Royal Society B: Biological Sciences. 2016;371(1688):20150110. doi: doi:10.1098/rstb.2015.0110.

8.	Meers J, Stout-Aguilar J, Nowakowski S. Sex differences in sleep health. In: Grandner MA, editor. Sleep and Health: Academic Press; 2019. p. 21-9.

9.	van der Schuur WA, Baumgartner SE, Sumter SR. Social media use, social media stress, and sleep: Examining cross-sectional and longitudinal relationships in adolescents. Health Communication. 2019;34(5):552-9. Epub 20180109. doi: 10.1080/10410236.2017.1422101. PubMed PMID: 29313723.

10.	van den Eijnden RJJM, Geurts SM, ter Bogt TFM, van der Rijst VG, Koning IM. Social media use and adolescents’ sleep: A longitudinal study on the protective role of parental rules regarding internet use before sleep. International journal of environmental research and public health. 2021;18(3):1346. PubMed PMID: doi:10.3390/ijerph18031346.

11.	Levenson JC, Shensa A, Sidani JE, Colditz JB, Primack BA. The association between social media use and sleep disturbance among young adults. Preventive Medicine. 2016;85:36-41.

12.	Cain N, Gradisar M. Electronic media use and sleep in school-aged children and adolescents: A review. Sleep Medicine. 2010;11(8):735-42. Epub 20100729. doi: 10.1016/j.sleep.2010.02.006. PubMed PMID: 20673649.

13.	Groff D, Sun A, Ssentongo AE, Ba DM, Parsons N, Poudel GR, et al. Short-term and long-term rates of postacute sequelae of SARS-CoV-2 infection: a systematic review. JAMA Network Open. 2021;4(10):e2128568-e.
